# Pralsetinib-Induced Lymphocytic Colitis

**DOI:** 10.14309/crj.0000000000001943

**Published:** 2026-01-05

**Authors:** Mauricio F. Jin, Sarah S. Tawfic, Xiao Jing (Iris) Wang

**Affiliations:** 1 Division of Internal Medicine, Mayo Clinic, Rochester, MN; 2 Division of Gastroenterology and Hepatology, Mayo Clinic, Rochester, MN

**Keywords:** pralsetinib, RET-selective tyrosine kinase inhibitor, lymphocytic colitis, microscopic colitis, drug-induced colitis, targeted therapy adverse events

## Abstract

Lymphocytic colitis (LC) is a subtype of microscopic colitis, often associated with chronic watery diarrhea and characterized by an increase in intraepithelial lymphocytes. Although various medications are implicated in drug-induced LC, its incidence as a treatment-related adverse effect of rearrangement during transfection-selective tyrosine kinase inhibitors is not known. We report a case of pralsetinib-induced LC in a patient with stage 3c nonsmall cell lung cancer. This case highlights pralsetinib-induced LC as a cause of persistent diarrhea that is refractory to standard antidiarrheal treatments and prompts gastrointestinal evaluation and histopathological examination to facilitate early diagnosis and management.

## INTRODUCTION

Pralsetinib is a rearrangement during transfection (RET) selective tyrosine kinase inhibitor (TKI) approved for treatment of metastatic nonsmall cell lung cancer (NSCLC) with RET-fusion and advanced/metastatic radioiodine-refractory thyroid carcinoma with RET-fusion.^1^ Although considered effective and safe in its treatment of these cancers, it is commonly associated with gastrointestinal (GI) treatment-related adverse events (TRAE).^1–3^ We report a unique case of pralsetinib-induced lymphocytic colitis (LC) in a patient treated for stage 3c NSCLC.

## CASE REPORT

A 71-year-old woman with no prior GI diagnoses was referred to GI clinic for persistent grade 3 diarrhea and biopsy-proven colitis during treatment with pralsetinib. She was started on pralsetinib 400 mg after work-up for thoracic back pain and dry cough revealed stage 3c NSCLC. Restaging computed tomography 2 months after initiating pralsetinib demonstrated near complete tumor response.

Three months after initiating pralsetinib, patient experienced lower abdominal cramping, anorexia, and persistent grade 3 diarrhea with occasional dark stools. Loperamide only minimally improved her diarrhea. Subsequent laboratory workup was unrevealing for infection. She underwent esophagogastroduodenoscopy and colonoscopy, notable only for mild scattered erythema in the rectosigmoid. Random colonic and rectal biopsies demonstrated an increase in intraepithelial lymphocytes with associated neutrophilic infiltrate and focal crypt abscess formation throughout the colon and rectum, consistent with LC. Her pralsetinib was held, and her diarrhea resolved 1 week later. However, restaging positron emission tomography-computed tomography demonstrated new uptake in the left lung concerning for tumor recurrence, and she was restarted on a reduced dose of pralsetinib 200 mg after holding for 2 weeks. Her dosage was increased to 300 mg 1 week after restarting pralsetinib, and she started experiencing grade 2 diarrhea. Pralsetinib was held again. She then received budesonide 9 mg for drug-induced LC, with resolution of her diarrhea in 1 day. She then restarted her pralsetinib 200 mg, with concomitant budesonide, increasing her dose to 300 mg 1 week later, with no recurrence of diarrhea.

## DISCUSSION

GI TRAE are known side effects of the Food and Drug Administration approved RET-selective TKIs, pralsetinib and selpercatinib. Reported GI TRAE include constipation, diarrhea, taste disorder, dry mouth, and lab abnormalities including elevated transaminases, alkaline phosphatase, and hyperbilirubinemia among others.^3^ However, the underlying mechanism for GI symptoms is not yet fully understood. To our knowledge, LC is not yet readily associated with RET-selective TKIs.

LC is a subtype of microscopic colitis, characterized by chronic, nonbloody, watery diarrhea with a normal appearing colon during colonoscopy and increased intraepithelial lymphocytes on histology.^4^ The pathogenesis of LC is complex, with data suggesting immunological, microbiomial, and genetic influences contributing to its development.^4^ Drug-induced LC is attributed to a wide range of commonly used medication classes, including but not limited to proton pump inhibitors, nonsteroidal anti-inflammatory drugs, selective serotonin reuptake inhibitors, to relatively less commonly prescribed medications such as checkpoint inhibitors and disease-modifying antirheumatic drugs.^4,5^ Discontinuation of the suspected offending agent is generally advised, but data to support this recommendation as the sole intervention are weak.^4^ Budesonide 9 mg daily for 6-8 weeks is the evidence-based standard of care for remission induction of microscopic colitis, with trials demonstrating clinical remission within 2 weeks of budesonide initiation.^4^ Moreover, long-term low-dose maintenance budesonide therapy may be required to maintain disease remission, as relapse rates after budesonide discontinuation is high.^6^

The exact mechanism by which RET-selective TKIs may cause LC is not clear. RET kinase is involved in regulating cellular proliferation, differentiation, and survival through various downstream signaling pathways, and disruption of these signaling pathways in GI epithelial cells could theoretically promote inflammatory gut responses leading to LC in individuals with predisposing genetic or microbiome compositions.^7^

Furthermore, diarrhea is a known TRAE of multitarget TKIs and many selectively targeted TKIs, such as the vascular epithelial growth factor receptor-selective TKI sunitinib and the ALK-selective ceritinib.^8–12^ Reports of colitis are sparse, and reports of microscopic colitis even rarer.^13,14^ It is theorized that VEGFR inhibition of intestinal angiogenesis leading to chronic mucosal ischemia may contribute to colitis.^15^ Pralsetinib and selpercatinib do exhibit very low potency VEGFR inhibition in comparison to cabozantinib and vandetanib, although the RET-selective TKIs' low potency questions whether standard dosages would drive this VEGFR-inhibition–theorized colitis or if another factor may be at play.^16,17^ Furthermore, diarrhea remains a common TRAE in VEGFR-sparing multikinase inhibitors.^18^ More studies are needed to further establish the connection between RET-selective TKIs and LC, perhaps aided by emerging second-generation RET-selective TKIs that exhibit even less off-target activity.^19^

The approval of RET-selective TKIs pralsetinib and selpercatinib marked significant advancements in precision oncology, providing more convenience and fewer side effects in comparison to previously used chemotherapy, radiation, and multitargeted TKIs. As the clinical application of RET-selective TKIs expands, understanding their safety profile and potential side effects becomes increasingly important.^20,21^ As in our case, management of RET-selective TKI-induced LC requires a multidisciplinary approach, but appropriate diagnosis and treatment can allow for continuation of an otherwise effective oncological option.

## DISCLOSURES

Author contributions: MF Jin contributed to case identification, data collection, literature review, manuscript drafting, and critical revision. SS Tawfic contributed to clinical case management, data interpretation, manuscript drafting, and critical revision. XJ Wang contributed to clinical case management, case interpretation, literature review, manuscript drafting, and critical revision. All authors meet ICMJE authorship criteria, approved the final manuscript, and agree to be accountable for all aspects of the work. X.J. Wang is the article guarantor.

Financial disclosure: None to report.

Previous presentation: Previously presented as a poster at the American College of Gastroenterology Annual Scientific Meeting, Philadelphia, PA, October 25–30, 2024.

Informed consent was obtained for this case report.

## ABBREVIATIONS:

GI: gastrointestinal
LC: lymphocytic colitis
NSCLC: non small cell lung cancer
RET: rearrangement during transfection
TKI: tyrosine kinase inhibitor
TRAE: treatment-related adverse event
VEGFR: vascular epithelial growth factor receptor
